# Efficacy of tranexamic acid supplemented with local infiltration analgesia in reducing blood loss in patients undergoing unicompartmental knee arthroplasty

**DOI:** 10.1038/s41598-023-44651-0

**Published:** 2023-10-12

**Authors:** Łukasz Wiktor, Bartłomiej Osadnik, Maria Damps

**Affiliations:** 1 Department of Trauma and Orthopaedic Surgery, Upper Silesian Children’s Health Centre, Medical University of Silesia, Katowice, Poland; 2 Department of Trauma and Orthopedic Surgery, SP ZSM Hospital, Chorzów, Poland; 3 Department of Anaesthesiology and Intensive Care, Upper Silesian Children’s Health Centre, Medical University of Silesia, Katowice, Poland

**Keywords:** Diseases, Outcomes research

## Abstract

This study aimed to investigate the efficacy of tranexamic acid supplemented with local infiltration analgesia in reducing blood loss in patients undergoing unicompartmental knee arthroplasty (UKA). This retrospective study was conducted on 176 individuals with a mean age of 64.27 (standard deviation [SD], 7.16) years undergoing unicompartmental cemented knee arthroplasty. The patients were divided into three groups according to patient blood management: I, patients without additional bleeding protocol (control group); II, patients intravenously administered tranexamic acid (TXA) (TXA group); and III, patients with exact TXA protocol combined with intraoperative local infiltration analgesia (LIA) (TXA + LIA group). Blood loss was measured as a substitute for blood loss by the maximal haemoglobin (Hb) drop compared with the preoperative Hb level. The mean Hb drops for the control, TXA, and TXA + LIA groups were 2.24 (16.0%), 2.14 (15.4%), and 1.81 (12.6%) g/dl, respectively. The mean hospitalisation days for patients in the control, TXA, and TXA + LIA groups were 5.91 (SD 1.24), 5.16 (SD 0.95), and 4.51 (SD 0.71) days, respectively. The combination of TXA with LIA reduces perioperative blood loss for patients after UKA.

## Introduction

Osteoarthritis is one of the most common causes of disability. Furthermore, treatment of the complications caused by osteoarthritis results in significant financial burden. Total knee arthroplasty (TKA), next to total hip arthroplasty (THA), is one of the most frequently performed elective orthopaedic surgeries. TKA mainly reduces pain, restores knee function, and improves patients’ quality of life. Unicompartmental knee arthroplasty (UKA) is performed on patients in whom only one knee compartment is affected by arthritis. UKA often involves the medial compartment of the knee. Compared with TKA, UKA is associated with preserving normal kinematics, translating into faster rehabilitation and quicker recovery. A simpler approach and the replacement of only one compartment are associated with minor blood loss. As UKA results in less postoperative pain, without blood transfusion, this procedure leads to shorter hospital stays and is less expensive than TKA^1–3^. The main contraindications to UKA are inflammatory arthritis, fixed varus deformity > 10°, fixed valgus deformity > 5°, range of motion < 90°, flexion contracture > 10°, contralateral meniscal damage or previous meniscectomy, tricompartmental arthritis, and grade IV patellofemoral chondropathy according to the International Cartilage Repair Society. Reducing perioperative blood loss is essential in patient blood management strategies. This approach involves evidence-based management to reduce the number of patients requiring postoperative transfusion after knee and hip replacement^4^. Tranexamic acid (TXA) is a fibrinolysis inhibitor that directly prevents plasminogen activation and inhibits the degradation of fibrin clots by activated plasmin. TXA promotes haemostasis and reduces the duration and quantity of blood loss^5,6^. Intravenous (IV) and topical TXA administration effectively reduces blood loss and risk of transfusion in patients after TKA^7,8^. Local infiltration analgesia (LIA) is a technique developed by Kerr and Kohan to reduce pain after TKA and THA^9^. It was initially assumed that ropivacaine, ketorolac, and adrenaline had infiltrated the tissues around the surgical field. LIA has been widely used for pain relief in patients undergoing total knee replacement for over 15 years. Blood loss can be evaluated either by estimating the loss or by measuring the drop in haemoglobin (Hb) and haematocrit (Hct) levels as a surrogate for blood loss^10,11^. This study aimed to investigate the efficacy of LIA-supplemented TXA in reducing blood loss in patients undergoing UKA.

## Materials and methods

We used the Strengthening the Reporting of Observational studies in Epidemiology protocol, which is designed for retrospective observational studies^12^. Patients diagnosed with unicompartmental osteoarthritis who underwent unilateral primary cemented medial UKA at ZSM Hospital in Chorzów between January 2018 and July 2022 were enrolled in this study. The following inclusion criteria were established for further evaluation: (1) patients undergoing medial UKA exclusively, (2) patients operated under spinal anaesthesia, and (3) patients operated on by one of the following two surgeons: Ł.W. and B.O. Patients (1) with pre-existing coagulopathy, (2) receiving long-term anticoagulant therapy before surgery, and (3) undergoing other additional procedures, including implant removal, were excluded. We established generally accepted contraindications for UKA, as described in the Introduction section. During the study period, only four patients underwent lateral UKA. Owing to a more extensive surgical approach and an incommensurably small number of patients, we excluded them from the analysis.

After considering the above criteria, 176 individuals were included for further analysis. The patients were divided into three groups based on patient blood management: I, patients without additional bleeding protocol (control group); II, patients intravenously administered 1.0 g of TXA 30 min before skin incision and 3 h later (TXA group); and III, patients with exact TXA protocol combined with intraoperative LIA (ropivacaine [100 mg] and epinephrine [0.25 mg] in a 50-ml solution) (TXA + LIA group). The differences in the number of individuals among the groups resulted from restrictions related to coronavirus disease 2019 during the study period and the growing number of UKA.

According to the Polish guidelines for preventing and treating venous thromboembolism, each patient received a dose of low-molecular-weight heparin (LMWH) 12 h before surgery (40 mg Clexane, Sanofi, Paris, France), and LMWH was continued postoperatively for 14 days. To assess blood loss, the Hb level before surgery was compared with the lowest Hb level during the hospital stay. Laboratory tests were performed on the day of hospital admission, the second postoperative day, and the day preceding discharge.

Follow-up for the included patients was extended to 1 month after discharge to assess the early readmission rate.

### Surgical technique

**Figure 1:**
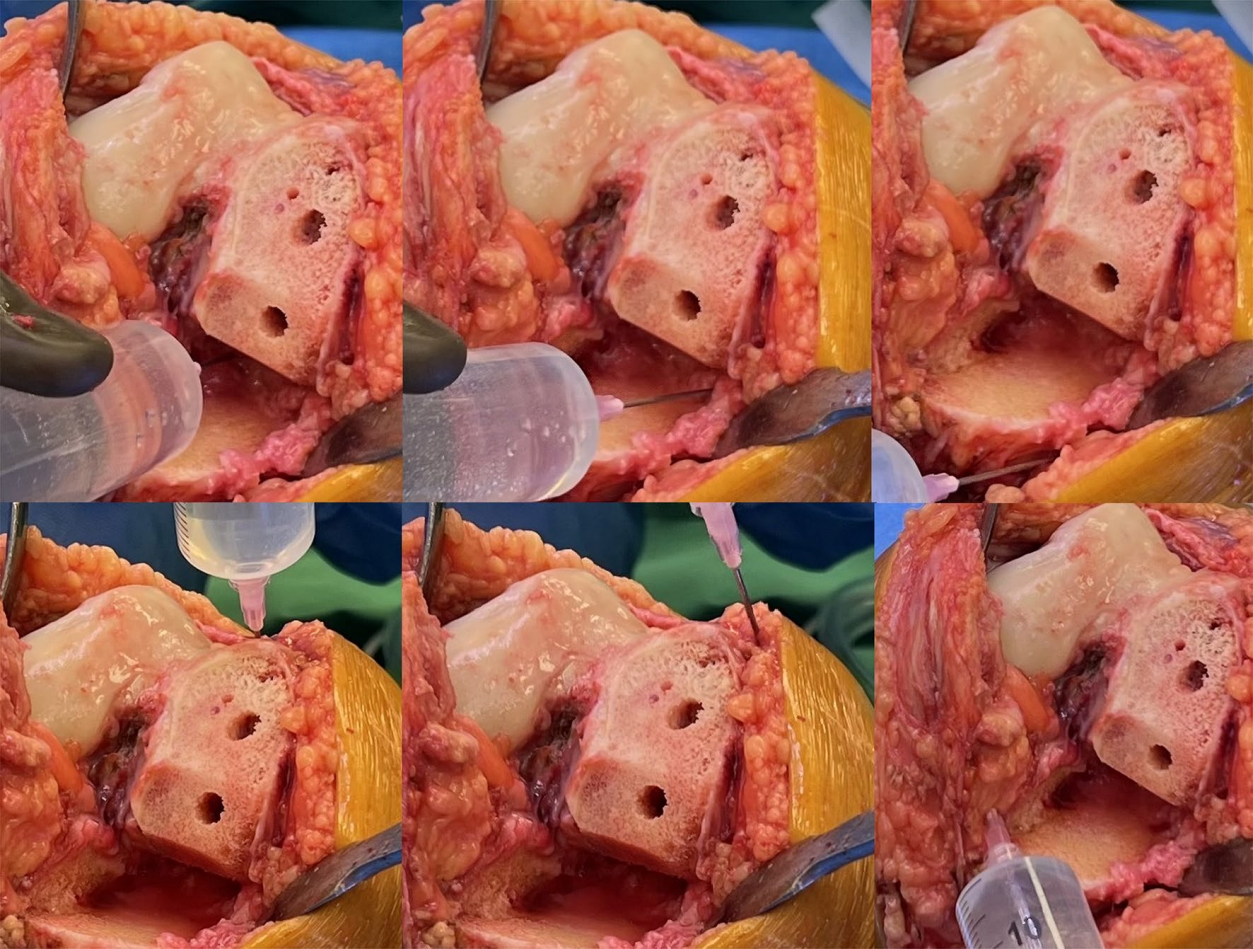
Intraoperative images showing LIA administration: posteromedial joint capsule, medial collateral ligament, supracondylar soft tissue, subcutaneous tissue and infrapatellar fat pad.

**Figure 2:**
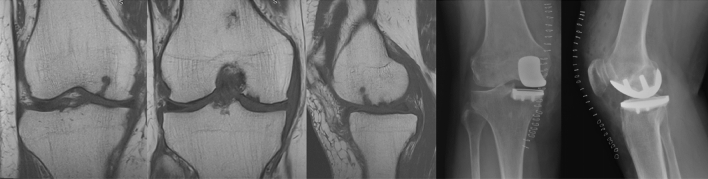
Magnetic resonance images, coronal and sagittal t1 scans show advanced medial compartment chondropathy. Postoperative radiographs after medial UKA, AP, and lateral view.

The surgeries were performed by two surgeons (Ł.W. and B.O.). A standard medial parapatellar approach was used in all the patients. Only one type of prosthesis was used in our study, JOURNEY™ UNI (Smith & Nephew, Cordova, USA). Surgical procedures were performed without a tourniquet, and thorough haemostasis was achieved. A drain was not used in all groups. The cement-receiving bone surfaces were cleaned using a pulse lavage system to remove fat residue, bone debris, marrow, and blood. The LIA solution contained 100 mg of ropivacaine and 0.25 mg of epinephrine in a 50-ml volume. According to the assumed intra-hospital protocol, half of the dose was used for TKA. The solution was sterile and prepared using a 50-ml syringe: 15 ml solution for the posteromedial joint capsule, 10 ml medial collateral ligament, 10 ml supracondylar soft tissue, and 15 ml subcutaneous tissue (Fig. 1). The joint capsule was tightly sutured using STRATAFIX Symmetric PDS Plus 1–0 (ETHICON, Johnson & Johnson, Germany). After the surgery, a compression dressing was applied to the operated limb. A standard rehabilitation programme was implemented on the first postoperative day. We present an example of a patient who underwent a medial UKA in Fig. 2.

This study was approved by the Bioethics Committee of Silesian Medical Chamber in Katowice, Poland (ŚIL.KB.1011.2022). All procedures in this study were in accordance with the ethical standards of the institutional and/or national research committee and with the 1964 Declaration of Helsinki and its later amendments or comparable ethical standards.

### Ethics approval and consent to participate

This study was approved by the Bioethics Committee of Silesian Medical Chamber in Katowice, Poland (ŚIL.KB.1011.2022). Informed consent was not required owing to the retrospective nature of the study.

## Results

A total of 176 (control group, 59; TXA group, 45; TXA + LIA group, 72) individuals were included in the analysis. All groups were homogeneous in terms of sex, with a female predominance (control group, 72.88%; TXA group, 75.55%; TXA + LIA group, 68.05%). The mean age of all patients was 64.27 (standard deviation [SD], 7.16) years. The mean body mass index (BMI) of all patients was 27.1 (SD, 1.88) kg/m^2^. Table 1 presents an overview of the study groups. In all groups, the overall blood transfusion rate after the primary UKA was 0%. We did not observe a drop in Hb < 9 g/dl in any patient.

**Table 1:** The overview of the study group.

	Control group	TXA group	TXA + LIA group
No	59	45	72
Age (mean)	65.37 (SD 7.87)	64.82 (SD 6.26)	63.02 (SD 7.15)
Sex (M/F)	16/43	11/34	23/49
BMI	27.34 (SD 1.95)	26.86 (SD 1.8)	27.07 (SD 0.71)
Hospital stay (day)	5.91 (SD 1.24)	5.16 (SD 0.95)	4.51 (SD 0.71)
Preoperative Hb (g/dl)	14.08 (SD 1.08)	13.9 (SD 1.05)	14.34 (SD 1.37)
Lowest Hb (g/dl)	11.84 (SD 1.22)	11.76 (SD 1.20)	12.57 (SD 1.35)
Maximum Hb loss (g/dl)	2.24 (SD 0.62)	2.14 (SD 0.82)	1.81 (SD 0.62)

### Statistical analyses

For statistical analyses, analysis of variance (ANOVA) parametric tests with Tukey’s post-test for unequal samples were used to assess the Hb drop, and the Kruskal–Wallis ANOVA test with Dun’s post hoc test was used to assess the length of hospitalisation. Spearman’s rho coefficients were used to determine correlations. All statistical tests were performed at p = 0.05.

**Figure 3:**
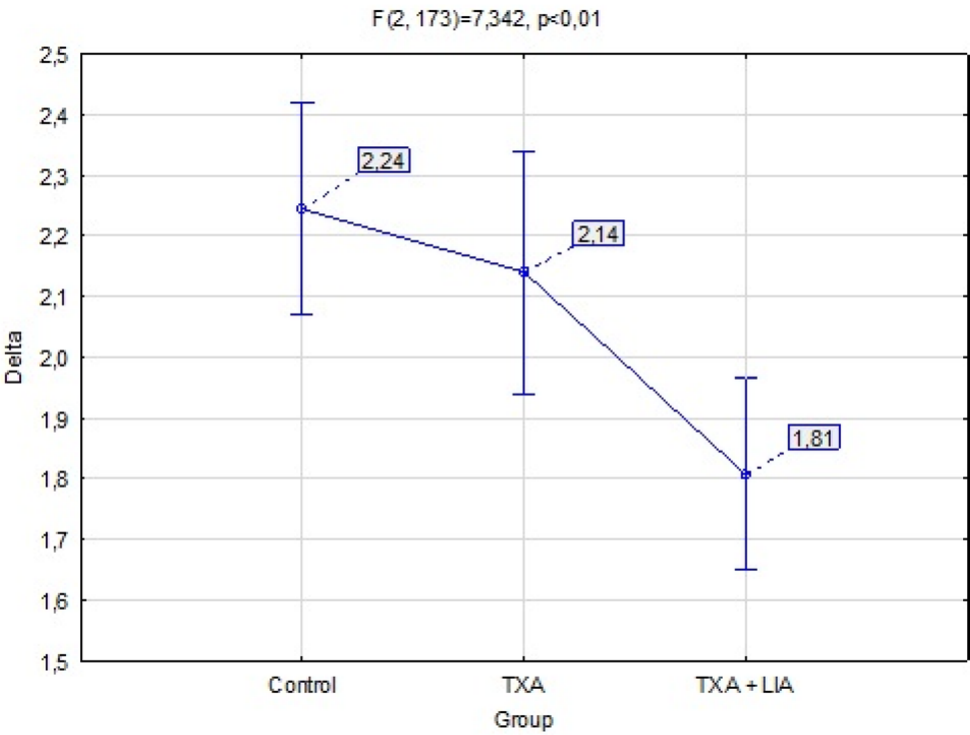
The plot of means post-op hemoglobin drop with a 95% confidence interval for the mean value by a group.

**Figure 4:**
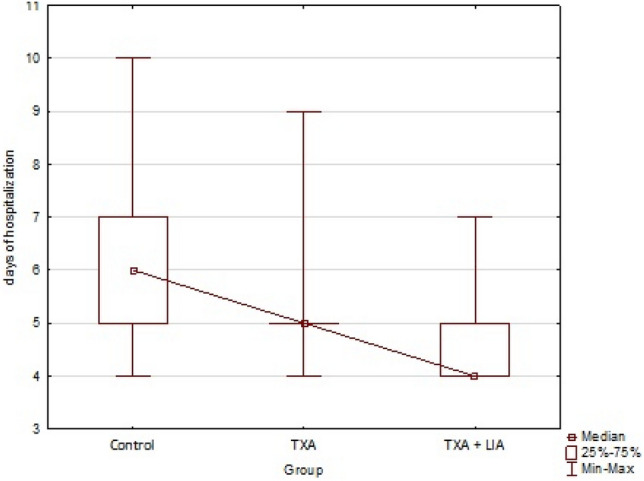
Box-whisker chart of the number of days of hospitalization by the group.

The TXA group had less Hb drop (M = 2.14 [15.4%]) than the control group (M = 2.24 [16.0%]), but the difference was not statistically significant (p = 0.749). In the TXA + LIA group, the decrease in Hb level was significantly (p = 0.001) lower (M = 1.81 [12.6%]) than that in the control group (M = 2.24 [16.0%]) and significantly lower (p = 0.043) than that in the TXA group (M = 2.14 [15.4%]). The mean values with 95% confidence interval are shown in Fig. 3. The length of hospitalisation was shorter (ME = 5) in the TXA group than in the control group (ME = 6), and the difference was statistically significant (p = 0.004). Moreover, the length of hospitalisation was significantly shorter (p < 0.001) in the TXA + LIA group (ME = 4.51) than in the control (ME = 6) and TXA (p = 0.005) groups. Figure 4 presents the statistics of the median, lower, and upper quartiles and minimum–maximum days of hospitalisation depending on the examined group. Based on Spearman’s rho correlation tests, sex and age were not significantly associated with a decrease in Hb levels. For the TXA (rho = 0.708, p < 0.001) and TXA + LIA (rho = 0.660, p < 0.001) groups, we observed a more significant decrease in Hb levels in patients with higher BMI than in those with lower BMI.

## Discussion

Different from TKA, UKA is not a surgical procedure related to an increased blood transfusion risk. The transfusion rates for patients undergoing UKA range from 0 to 7.4%^10,13–15^. Less blood loss in UKA is mainly due to the less invasive approach, limited bone cuts to a single compartment, and lack of the need for extensive soft tissue balancing. IV and topical TXA administration effectively reduces blood loss and transfusion risk in patients after TKA^7,8,16^. The benefits of TXA in decreasing blood loss in UKA have yet to be fully validated. Topical TXA reduces perioperative blood loss in patients undergoing UKA without associated complications^17,18^. However, there have been concerns regarding the chondrotoxicity of TXA in UKA, where native cartilage is still present in the knee after surgery^19,20^. Therefore, some researchers do not recommend using TXA in UKA^20^. Pongcharoen et al.^21^, on a group of 120 knees, demonstrated that using IV TXA in UKA resulted in lower blood loss than not using IV TXA in UKA, but the differences were not significant. Similarly, in our study, the TXA group had less Hb drop than the control group, but the difference was not statistically significant (p = 0.749). Our study is the first to determine the effects of combining TXA with LIA on blood loss after UKA. The decrease in Hb level was significantly lower (p = 0.001) in the TXA + LIA group than in the control and TXA (p = 0.043) groups.

We have started using LIA routinely in TKA and UKA since January 2021. Contrary to most recommendations, we used a volume of 100 ml for TKA and 50 ml for UKA because high LIA volumes may increase the risk of prolonged wound drainage or leaking^9,22^. Patients in the LIA group had significantly lower pain scores according to the visual analogue scale, especially in the first 2 postoperative days, compared with other patients. However, this detailed assessment differs from the purpose of the present study. We did not use a drain for any of the UKA groups. Surgical drainage increases postoperative blood loss^23,24^.

Several authors have assessed the risk factors for morphological decrease after TKA and THA to identify patients at risk for blood transfusion^25,26^. Preoperative anaemia is the most significant risk factor for blood transfusion after TKA^27^.

None of the patients in our study required a transfusion. According to in-hospital guidelines, surgery is delayed in patients with anaemia until their Hb values are equalised. BMI, female sex, and age are significant predictors of transfusion in patients undergoing elective TKA^28–30^. According to our analysis, based on Spearman’s rho correlation tests, sex and age were not preoperative predictors of higher blood loss rates. For the TXA (rho = 0.708, p < 0.001) and TXA + LIA (rho = 0.660, p < 0.001) groups, we observed a more significant decrease in Hb levels in patients with higher BMI than in those with lower BMI. In recent years, outpatient and fast-track UKA pathways have gained increasing attention^31–33^. Sephton et al.^34^, in line with the results of our study, reported a transfusion rate of 0% in patients after UKA. However, the average hospitalisation time in their study was shorter (3.3 days) than that of our study. We did not follow fast-track UKA in our study. The length of hospitalisation was shorter in the TXA (p = 0.004) and TXA + LIA groups (p < 0.001) than in the control group. Moreover, Sephton et al. concluded that postoperative control of Hb and Hct was unnecessary and could be omitted in postoperative procedures.

Although patients undergoing knee replacement routinely receive prophylactic anticoagulants, venous thromboembolic events continue to occur. In our study, we used a double dose of 1.0 g TXA, administered 30 min before skin incision and 3 h later, and we did not observe any case of deep venous thrombosis or pulmonary embolism.

The strength of our study is the homogeneity of the comparison groups. Based on the data analysis, we confirmed the synergistic effect of TXA and LIA in reducing perioperative blood loss. However, this study has some limitations. The main limitation of this study was its retrospective design. Furthermore, we did not evaluate perioperative blood loss.

Further analysis is required to understand the effects of combining TXA with LIA in reducing perioperative blood loss and blood transfusion rate after unicompartmental knee replacement. Assessing the predictive risk factors for UKA is difficult because of the low transfusion rate and requires a large cohort of patients to reach meaningful conclusions.

## Conclusions

The combination of TXA and LIA reduces perioperative blood loss in patients after UKA. The use of LIA in the perioperative protocol does not increase the risk of local wound healing complications. The use of TXA with LIA results in shorter length of hospitalisation compared with the use of isolated TXA.

## Data Availability

The data generated and analyzed in the current study are available from the corresponding author on reasonable request.

## Abbreviations

ANOVA: Analysis of variance
BMI: Body mass index
Hb: Haemoglobin
Hct: Haematocrit
LMWH: Low-molecular-weight heparin
SD: Standard deviation
THA: Total hip arthroplasty
TKA: Total knee arthroplasty
TXA: Tranexamic acid
UKA: Unicompartmental knee arthroplasty
